# A comparison of isolated circulating tumor cells and tissue biopsies using whole-genome sequencing in prostate cancer

**DOI:** 10.18632/oncotarget.6330

**Published:** 2015-11-05

**Authors:** Runze Jiang, Yi-Tsung Lu, Hao Ho, Bo Li, Jie-Fu Chen, Millicent Lin, Fuqiang Li, Kui Wu, Hanjie Wu, Jake Lichterman, Haolei Wan, Chia-Lun Lu, William OuYang, Ming Ni, Linlin Wang, Guibo Li, Tom Lee, Xiuqing Zhang, Jonathan Yang, Matthew Rettig, Leland W.K. Chung, Huanming Yang, Ker-Chau Li, Yong Hou, Hsian-Rong Tseng, Shuang Hou, Xun Xu, Jun Wang, Edwin M. Posadas

**Affiliations:** ^1^ Beijing Genome Institute-Shenzhen, Shenzhen 51803, China; ^2^ Urologic Oncology Research Program, Cedars-Sinai Medical Center, Los Angeles, CA 90048, USA; ^3^ Department of Statistics, University of California, Los Angeles, CA 90095, USA; ^4^ Institute of Statistical Sciences, Academia Sinica, Taipei 11529, Taiwan; ^5^ Department of Molecular and Medical Pharmacology, University of California, Los Angeles, CA 90095, USA; ^6^ Department of Pathology and Laboratory Medicine, University of California, Los Angeles, CA 90095, USA; ^7^ California NanoSystems Institute, University of California, Los Angeles, CA 90095, USA; ^8^ The Guangdong Enterprise Key Laboratory of Human Disease Genomics, BGI-Shenzhen, Shenzhen 51803, China; ^9^ Departments of Medicine and Urology, University of California, Los Angeles, CA 90095, USA; ^10^ Department of Medicine, Division of Hematology-Oncology, VA Greater Los Angeles Healthcare System, Los Angeles, CA 90073, USA; ^11^ Princess Al Jawhara Centre of Excellence in Research of Hereditary Disorders, King Abdulaziz University, Jeddah 21589, Saudi Arabia; ^12^ James D. Watson Institute of Genome Sciences, Zhejiang University, Hangzhou 310058, China; ^13^ Department of Biology and the Novo Nordisk Foundation Center for Basic Metabolic Research, University of Copenhagen, Copenhagen 1599, Denmark

**Keywords:** circulating tumor cell, prostate cancer, whole genome sequencing, liquid biopsy, cancer heterogeneity

## Abstract

Previous studies have demonstrated focal but limited molecular similarities between circulating tumor cells (CTCs) and biopsies using isolated genetic assays. We hypothesized that molecular similarity between CTCs and tissue exists at the single cell level when characterized by whole genome sequencing (WGS). By combining the NanoVelcro CTC Chip with laser capture microdissection (LCM), we developed a platform for single-CTC WGS. We performed this procedure on CTCs and tissue samples from a patient with advanced prostate cancer who had serial biopsies over the course of his clinical history. We achieved 30X depth and ≥ 95% coverage. Twenty-nine percent of the somatic single nucleotide variations (SSNVs) identified were founder mutations that were also identified in CTCs. In addition, 86% of the clonal mutations identified in CTCs could be traced back to either the primary or metastatic tumors. In this patient, we identified structural variations (SVs) including an intrachromosomal rearrangement in chr3 and an interchromosomal rearrangement between chr13 and chr15. These rearrangements were shared between tumor tissues and CTCs. At the same time, highly heterogeneous short structural variants were discovered in *PTEN*, *RB1*, and *BRCA2* in all tumor and CTC samples. Using high-quality WGS on single-CTCs, we identified the shared genomic alterations between CTCs and tumor tissues. This approach yielded insight into the heterogeneity of the mutational landscape of SSNVs and SVs. It may be possible to use this approach to study heterogeneity and characterize the biological evolution of a cancer during the course of its natural history.

## INTRODUCTION

Circulating tumor cells (CTCs) are rare cancer cells within the circulation in patients with an underlying solid tumor malignancy [1]. We and other have postulated that they play a role in the process of metastases [2] and, as such, may be an alternative source of tissue for molecular study- i.e. a “liquid biopsy”. Such an approach would confer an important advantage over conventional tissue biopsy: characterization of the dynamic biology of a cancer. Detection and characterization of CTCs has been technically challenging due to their extremely low relative abundance in the bloodstream [3, 4]. Many assays [5–13] efficiently capture CTCs, but do not provide cells appropriate for contemporary molecular analyses such as whole genome sequencing (WGS).

Several studies have shown similarities between CTCs and tumor tissues using targeted sequencing approaches [14–16] and copy number analysis [17]. Our group and others have performed whole exome sequencing (WES) on CTCs [18–20]. While an important advance, the coverage of the sequencing (43–71%) could still be improved. In addition, WES has its intrinsic limitations to identify genome rearrangements, which are now recognized as important in prostate cancer (PCa) [21].

With advances in single-cell sequencing [22, 23] and single-CTC isolation technologies [15, 18], our group set out to compare single-CTCs and tumor biopsies using WGS. We demonstrated that high quality WGS can be done utilizing these single-CTCs. This improved quality in sequencing allowed us to test the hypothesis that CTCs and tumor tissues have shared somatic single nucleotide variations (SSNVs) and rearrangements due to their shared biological nature.

## RESULTS

### Patient information and CTC retrieval

The studied patient underwent a prostatectomy and then experienced biochemical relapse 1 year later. He was started on medical castration therapy. He progressed through castration therapy 3 years later. Multiple osseous metastases were detected at that time. He then underwent multiple hormonal maneuvers but developed hepatic metastases. He received multiple systemic therapies including abiraterone, docetaxel, and an experimental targeted therapy. Commercially-available genomic profiling of a biopsy from his hepatic metastasis was performed but did not identify any targetable mutations.

**Figure 1:**
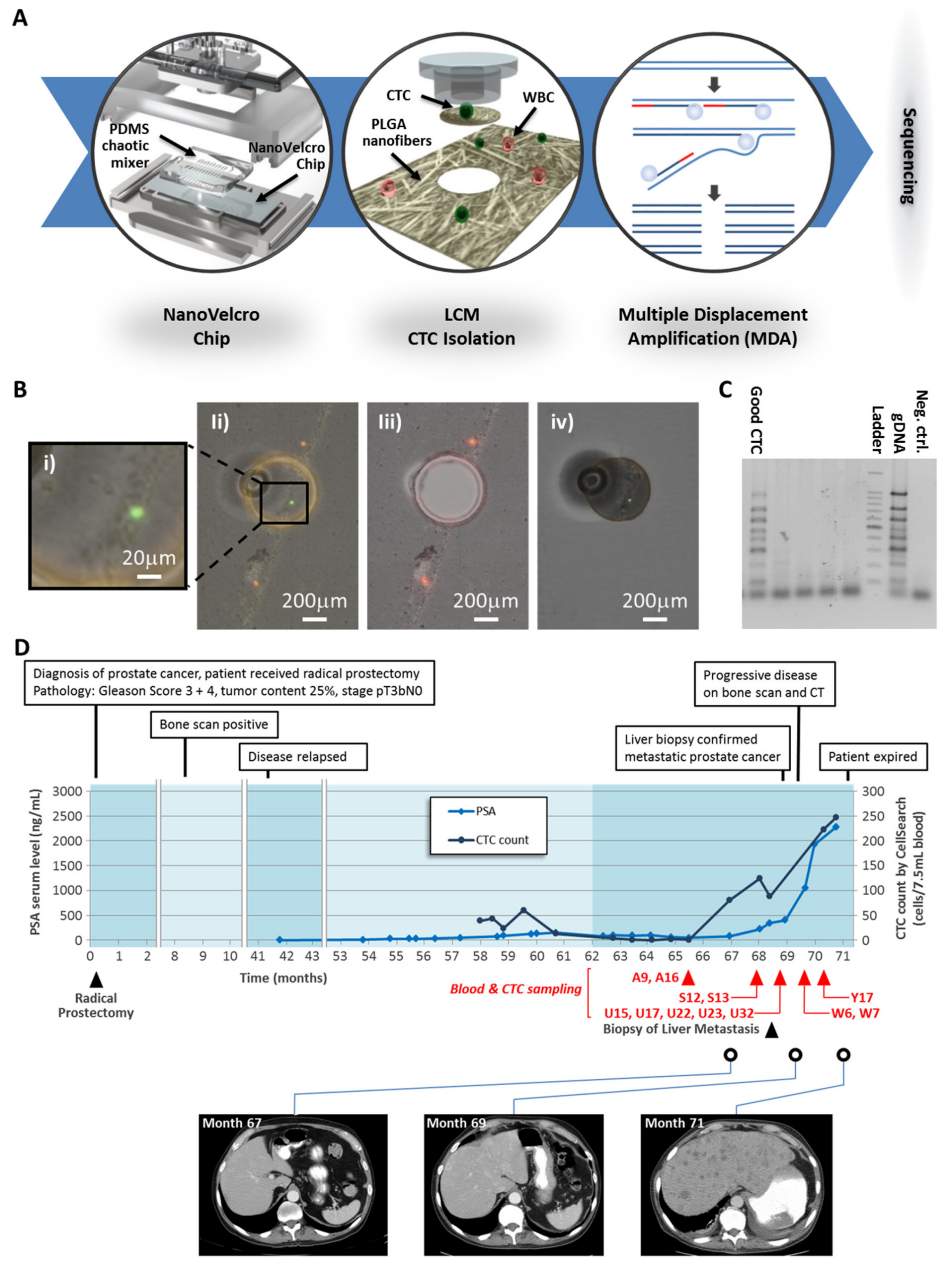
Performing CTC WGS in a patient with lethal liver metastasis **A.** Patient history and sampling time. **B.** Schematic workflow for our CTC WGS technology. (i) The NanoVelcro Chip consists of a cell-affinity substrate electrospun with PLGA nanofibers and an overlaid PDMS chaotic mixer. (ii) After determining CTCs based on their fluorescence (CD45-/CK+) and morphology, the LCM microscope was used to isolate single CTCs by laser dissection. (iii) Schematic illustration of WGA using multiple displacement amplification. (iv) Whole genome sequencing. **C.** Micrograph images recording the process of CTC isolation by LCM. (i) CTC identification; (ii) UV laser dissection; (iii) The CTC is removed from the substrate; (iv) isolated CTC was confirmed on the LCM cap. **D.** A gel electrophoresis figure showing good amplification and poor amplification WGA products.

NanoVelcro Chip was used in conjunction with a laser capture microdissection (LCM) microscope to isolated the captured CTCs (Figure 1A and 1B) [18]. We isolated a total of 99 CTCs from 5 blood samples collected over 4 months. A previous verified multiplex PCR of 8 housekeeping genes was performed to assess the integrity of the amplified DNA (Figure 1C) [22]. High-quality DNA samples suitable for WGS were obtained from 2 CTCs captured in at the outset of our collections (A9 and A16) and 2 from a visit 4 months later (U15 and U17). Moderate-quality DNA samples suitable for WES were obtained from an additional 8 CTCs (S12, S13; U22, U23, U32; W6, W7, Y17). White blood cells (WBCs) and adjacent normal prostate tissue obtained from his archival prostatectomy specimen were used as controls. A time course of his clinical history with specimen collection is presented in Figure 1D and supplementary Figure S1.

### Single-CTC WGS was performed with quality comparable to tissue sequencing

**Figure 2:**
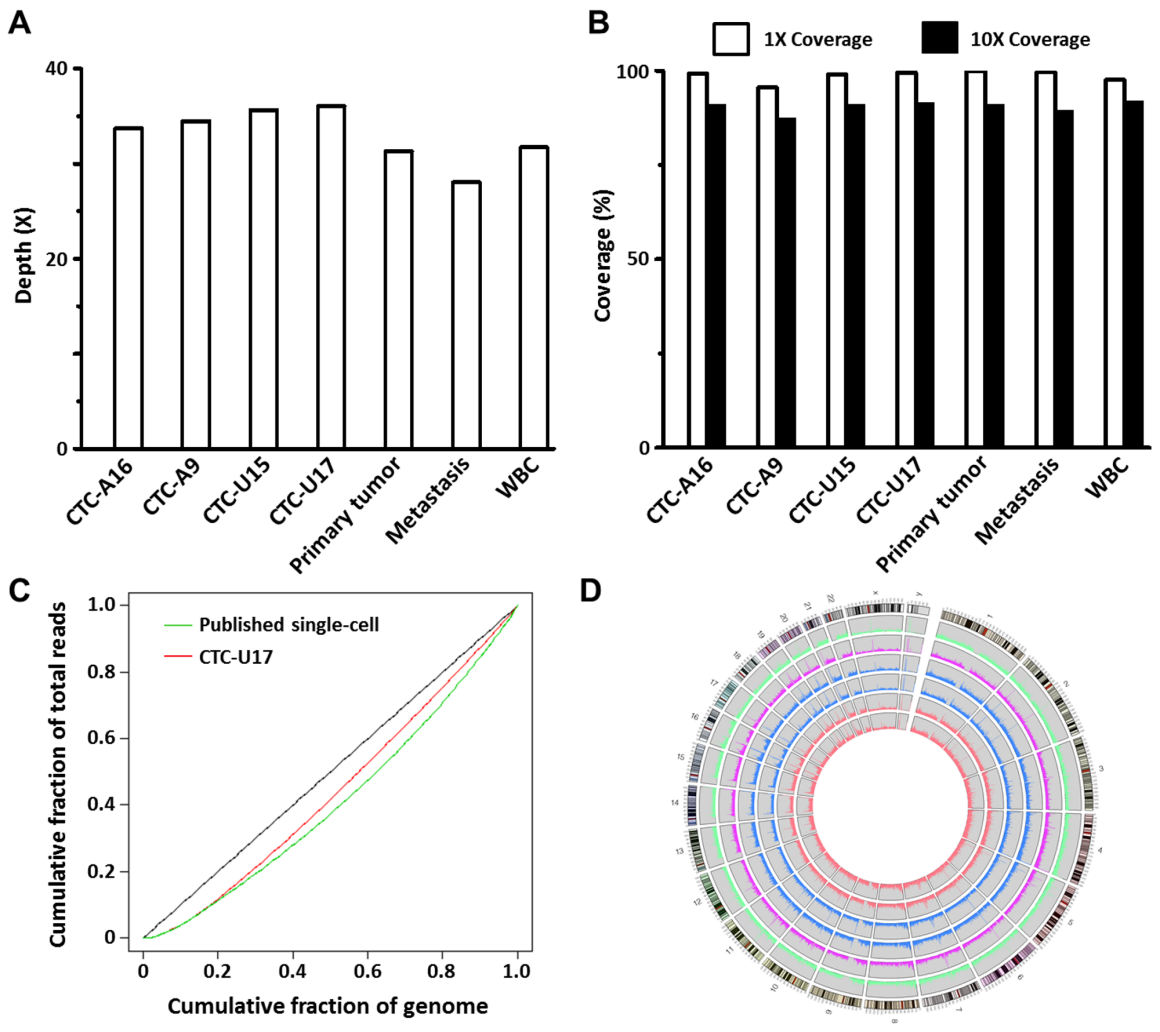
Sequencing quality assessments **A.** The sequencing depth of each sample. **B.** The percentage of area covered. **C.** The Lorenz curves comparing the homogeneity of coverage of CTC-U17, and a published single-cell sequencing data. **D.** The SNP densities of CTCs and tumor tissues. Height of rectangles ranges from 0–1000/100 Kb, bin = 100 Kb. The rings from the inside out represent CTC-U17, CTC-U15, CTC-A9, CTC-A16, liver metastasis, and primary prostate tumor. The outermost ring represents the karyotype of the human genome reference sequence (hg19), with red areas being centromeres.

WGS was carried out with paired-end 100-bp reads and ~350 bp inserts on an Illumina platform. Mean depths of 28–36X were achieved. All of the tumor and CTC sequencing runs had more than 95% genome coverage (95.6% to 99.6%) with more than 87% (87.6% to 91.5%) of the areas covered in sufficient depth (>10X) for calling variants with high confidence (Figure 2A and 2B; supplementary Table S1). GC content was compared between single CTCs and the WBC control to asses WGA quality. Correlation coefficients were all > 0.999, which indicated the absence of WGA associated bias (supplementary Table S2). Uniformity of coverage was assessed by Lorenz curves as proposed by a previous single cell sequencing study [24]. Our single-CTC WGS coverage uniformity was similar to the published date of the aforementioned study (Figure 2C). The single CTC coverage uniformity was also similar to that seen in WBC and primary tumor tissue WGS (supplementary Figure S2). Finally, the comparison of single nucleotide polymorphisms (SNPs) also showed that the total number and the distributions of SNPs in single-CTC samples were not different from the tumor tissues (Figure 2D and supplementary Table S3). In summary, all results were consistent with high-quality sequencing.

### SSNV comparison between CTCs and tumor biopsies

**Figure 3:**
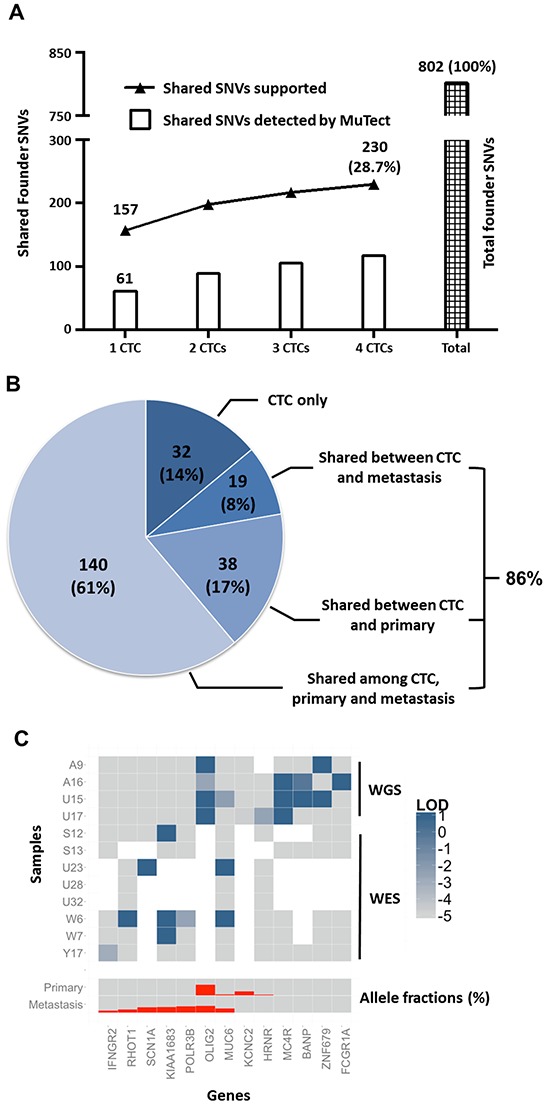
The similarity of SNVs between CTCs and tumor tissue **A.** The number of founder SSNVs (SNVs shared between primary and metastatic tumors) discovered when a different number of CTCs are involved in the analysis. The upper curve represents the number of shared SNVs identified by the presence of supporting reads. **B.** The origin of clonal SSNVs in CTCs. **C.** The exonic mutational landscape of this patient's CTCs. Higher log odd (LOD) scores indicate more likely the CTC harboring the mutation. For the concern of visualization, we used base 10 log and truncated the maximum value to 1. Thus, LOD = 1 means the posterior probability of a mutant is 10 times as big as the posterior probability of normal and LOD ≥ −5 means the likelihood of mutant is greater than normal (see Methods for details). The allele fractions of the primary and metastatic tumors are also plotted.

SSNV profiles of CTCs to the primary and metastatic tissues were compared using the MuTect algorithm [25]. We anticipated that some genomic alterations would not be present in all clones given the underlying heterogeneity within the population of tumor cells [26]. As such, we first identified the founder SSNVs shared between the primary and metastatic tumors. These founder SSNVs were then used to assess the similarity between CTCs and tissues. Among the 802 founder SSNVs in primary and metastatic PCa cells, a median of 61 (range 43–74) mutations were identified by MuTect in each of the single-CTCs. However, with a manual review of founder mutation sites, a median of 157 mutations (range 125–177) were identified with supporting reads in the single-CTCs. The identified shared founder SSNVs increased when the size of the CTC pool increased. A total of 230 founder SSNVs (28.7%) had supporting reads discovered in any of the four CTCs, where 117 SSNVs were called by MuTect (Figure 3A). To further evaluate the similarity between CTCs and tumor tissues, we identified clonal SSNVs from CTCs—defined as SSNVs shared by more than three CTCs. These SSNVs were repeatedly detected in more than three single-cell sequencing runs and can be considered high confident mutations, as previously reported [20, 22]. We found that 86.0% (197/229) of these clonal SSNVs in CTCs had supporting reads discovered in either the primary or metastatic tissues, where 116 SSNVs (50.7%) were called by MuTect (Figure 3B).

To further examine the heterogeneity of the CTC mutational landscape, WES was performed on 8 of the CTCs with moderate quality DNA. Coverage of 24.6–78.5% was achieved. To assess the probability of mutations in the single CTCs at each locus based on the sequencing reads, we derived the log odd (LOD) score using a Bayesian model adjusted by the allele drop out (ADO) rate (see detailed methods in the supplementary information). A heatmap was constructed based on the LOD score of 13 exonic mutations that were detected either in at least two CTCs or at least one CTC and a tumor tissue sample. Additional exonic SSNVs were found shared between CTC and tumor tissues in the WES study, which were not detected in the CTC WGS (Figure 3C). To better evaluate if the clonal mutations are more frequently found in CTCs, we plotted the allele fractions of each exonic mutations in the primary and metastatic tumors. We observe that mutations in primary or metastasis with higher allele fractions, which are more clonal, are more frequently mutated in CTCs. These findings were confirmed using three additional methodologies. First, we compared our tumor tissues and CTCs with both WBC and normal adjacent prostate tissue instead of using a more general approach of comparing tumor tissue with one normal tissue source to identify SSNVs. Second, we performed WES on a separate batch of WBCs and confirmed that 99.1% (770/777) of the exonic SSNVs detected in our WGS were not germline SNPs. Third, we randomly selected SSNV loci for additional confirmation by Sanger sequencing. The majority (23/25, 92%) of them were confirmed (supplementary Table S4).

### Structural variation (SV) comparison between CTCs and tumor biopsies

**Figure 4:**
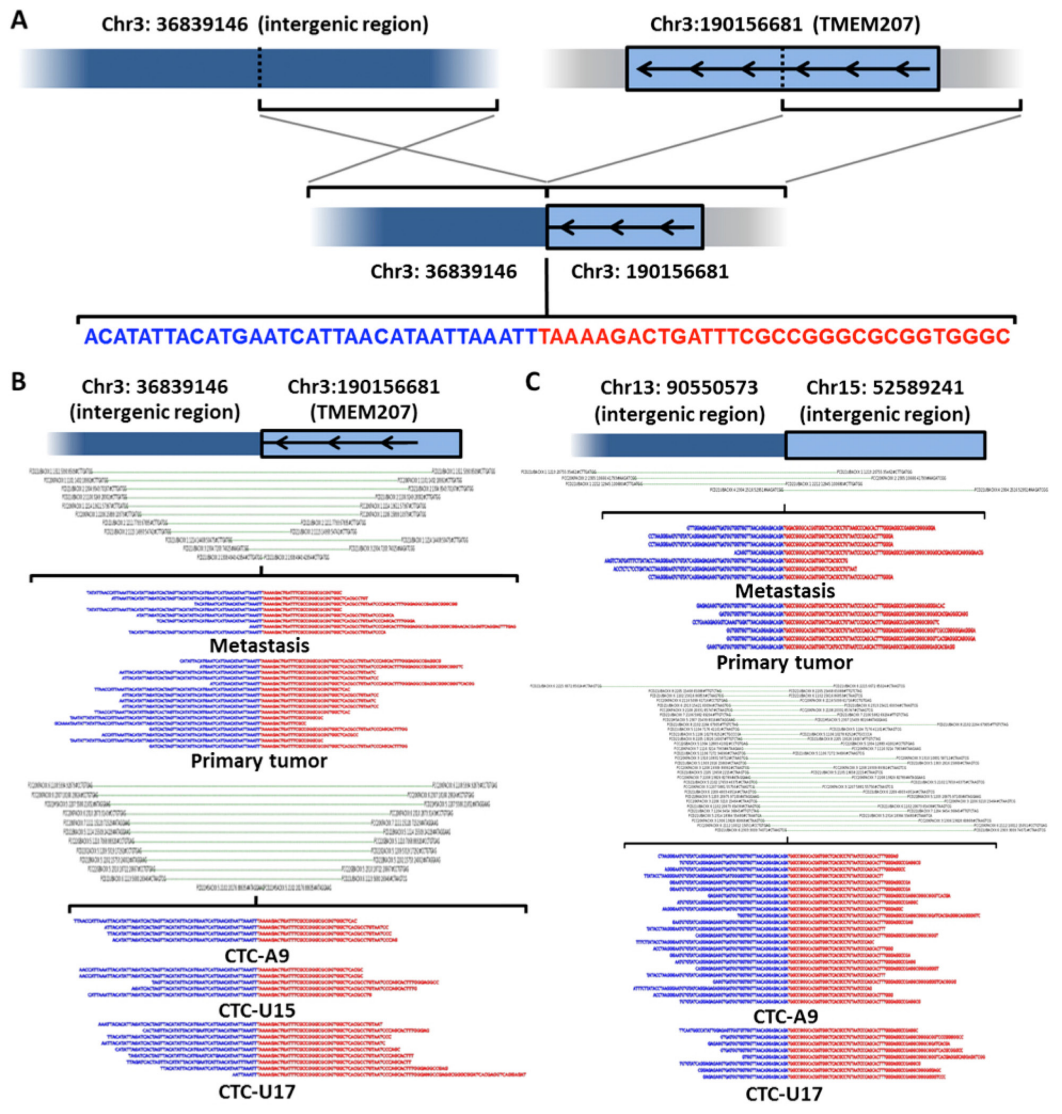
Shared SVs between CTCs and tumor tissues **A.** Illustrative representations of the TMEM207 rearrangement. **B.** The breakpoint and supporting reads of the TMEM207 rearrangement in primary, metastatic tumors and CTCs (A9, U15 and U17). **C.** The breakpoint and supporting reads of a chr13-chr15 translocation in CTCs, indicating exactly the same rearrangement in primary, metastatic tumors and CTCs (A9, and U17).

To examine the similarities in SVs, we utilized a two-step approach employing the CREST algorithm [27] to identify breakpoints and then performed manual checks of supporting reads in multiple samples. We found no rearrangements associated with ERG, ETS, or their reported fusion partners [28] in the CTCs or tumor tissues. Two SVs were shared among CTCs and both tissues, including an intrachromosomal SV in chromosome 3 (Figure 4A and 4B) and a SV between chromosome 13 and 15 (Figure 4C). The SV in chromosome 3 involves TMEM207, which has been associated with migration and invasiveness [29]. The breakpoints of these SVs are identical between cancer tissues and the CTCs.

**Figure 5:**
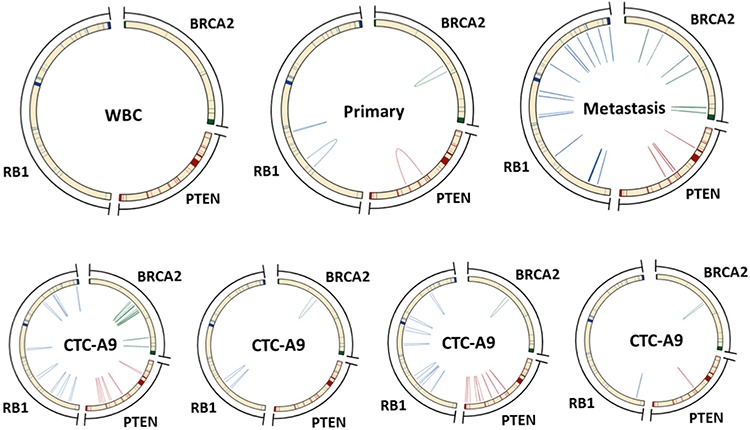
SVs in important tumor suppressor genes were shared between CTCs and tumor tissues Heterogeneous SVs were found in important tumor suppressor genes, including *BRCA2*, *PTEN* and *RB1*. These SVs were mostly between 0.6 kb to 2 kb in size, and were not detected in the WBCs and normal tissue controls. The patterns varied widely between primary and metastasis as well as each of the CTCs.

Finally, we screened known tumor suppressors for possible functional SVs. We found complex SV profiles in *PTEN*, *RB1* and *BRCA2* in all our samples that were not detected in the WBCs and normal tissue controls (Figure 5). These SVs were mostly between 0.6 kb to 2 kb in size. The patterns varied widely between primary and metastasis as well as each of the CTCs.

### Copy number variation (CNV) comparison between CTCs and tumor biopsies

CNVs represent important genomic alterations in PCa [30]. CNVs were assessed by the CASS and FREEC algorithms [31, 32]. No CNVs were identified in the primary tissue. This finding was confirmed by an array comparative genomic hybridization (aCGH) assay, suggesting that no founder CNVs were present in this patient (supplementary Figure S3)—a common finding in PCa patients [33]. In the liver metastasis sample, we observed some large segment amplifications, including 8q, which are also commonly reported in PCa [34]. Besides the small deletions near the ends of the chromosomes or close to the centromere, the single-CTC WGS data did not identify any prominent CNVs. The similarity between CTCs and tumor tissues by CNV analysis was limited due to the lack of founder CNVs. However, this result does demonstrate a highly homogeneous WGA process for our single CTCs (supplementary Figure S4).

## DISCUSSION

PCa continues to be the most common cancer affecting men in the United States. Over 20,000 American men will die from this disease in 2015 [35]. The presence of evolving temporo-spatial heterogeneity in PCa has been recognized as an important biological problem with clinical impact [33, 36]. Tumor tissue analysis (typically from biopsy or resection) remains the most commonly employed means of studying metastatic PCa in patients. Bone is the most common site for PCa metastasis. Though possible to obtain, bone biopsies are invasive and often technically challenging. Moreover, it is difficult to obtain biopsy at a clinically optimal frequency (e.g. monthly) for patients with advanced disease. Given the pace of disease progression in certain patients, it may be necessary to have this dynamic range if we attempt to understand the evolving biology underlying the disease. As such, CTCs are an appealing alternative source of information if the underlying biology of the malignancy is reflected within them.

This study shows that it is possible to obtain high-quality WGS data from individual CTCs following a microfluidic-based isolation approach. Specifically, we used a unique combination of technologies: the NanoVelcro chip for CTC capture, LCM for single-CTC isolation, and an Illumina sequencer for WGS. Our methodology achieved WGS coverage of above 95% across the 4 single-CTCs. All quality assessments from the single-CTC WGS were comparable to that obtained from tissue sequencing. This made it possible to analyze SSNVs and SVs in CTCs and conduct a comparison with tumor tissue from a PCa patient.

Our high-level of coverage was attributed to the unique advantages of our combined methodology over other existing CTC isolation and sequencing technologies [19, 20]. First, highly efficient CTC enrichment was achieved by combining NanoVelcro substrates and a microfluidic chaotic mixer as described previously [37, 38]. Second, using LCM, single-CTCs immobilized on the NanoVelcro substrates were visually distinguished from the surrounding WBCs and were manually and specifically isolated. Third, the negatively charged PLGA NanoVelcro substrates prohibited non-specific adsorption of cell-free DNA, and decreased contamination by circulating DNA in the subsequent WGS [39]. The integrity of the CTC DNA was preserved by this streamlined protocol that allowed rapid processing from the initial CTC enrichment steps to WGA [18]. Instead of PCR-based amplification, multiple displacement amplification (MDA) was utilized to amplify CTC DNA in long fragments. This approach decreased the loss of unamplified segments. MDA is also the least biased approach that reduces false positive SSNVs [40]. Knowing that paraformaldehyde fixation compromises DNA quality, we intentionally avoided the use of paraformaldehyde for the CTCs to minimize interference during MDA [41]. An additional rigorous quality check was employed to select high-quality CTC samples by a multiplexed PCR based on 8 housekeeping genes. With these technological improvements, the quality of our WGS exceeded all previously published attempts to sequence CTCs.

The high level of coverage in our WGS enabled comparison of SSNVs in CTCs and tumor tissues. This created the opportunity to perform a comprehensive comparison of SSNVs at the whole genome level. Comparing 4 single CTCs with tumor tissues from the index patient, we found that 86% of the clonal SSNVs identified in the CTCs (Figure 3B) were shared with the primary or metastatic tumor tissues. This observation is supported by a recent work by Lohr et al., which revealed 70% of the clonal SSNVs in CTCs can be traced back to tumor tissues using WES [20]. Due to the concern that some of the shared SSNVs were germline SNPs not detected in the control tissues, rigorous standards were applied for the identification of SSNVs. WGS was performed on both WBCs and the adjacent normal prostate tissue to increase the sensitivity of the germline SNP detection. Additional WES on a separate batch of WBCs confirmed that 99.7% of the exonic SSNVs detected in our WGS were not present in WBCs as germline SNPs. This demonstrates that a minimal level of germline SNPs was present and therefore could not confound our analyses. Further Sanger sequencing on randomly selected loci validated 92% of the SSNVs identified in our study.

There remained a concern that some of the shared mutations between CTCs and tissues were introduced during single-cell DNA amplification and sequencing. To avoid this error, the comparison of SSNV was based on WGS data obtained from the primary and metastatic tumor tissues. We identified a total of 802 founder SSNVs shared between the primary and metastatic tumors. Then we determined if these SSNVs were present in CTCs. In the literature, the established MDA-induced sequencing error rate is in the range of 10^−5^ [22, 23]. As such it is unlikely that a significant portion of these shared mutations (28.7%) were caused by WGA errors.

In this study, we identified 9 exonic SSNVs shared between CTCs and either of the tissues, (Figure 4C) as well as more than 200 somatic genomic SSNVs shared between CTCs and both of the tumor tissues. Compared to the report showing as many as 73% of SSNVs shared between primary tumor and metastasis via WES [20], we noted only a minor portion (28.7%) of tumor tissue SSNVs in the CTCs using WGS approach. This result was not unexpected considering the known heterogeneity in PCa as well as the clinical presentation of the analyzed patient. A high degree of intratumoral genomic heterogeneity in cancer has been shown by multiple-region exome sequencing on multiple clones obtained from a solid tumor [26]. This same approach in PCa also showed a small number (0–8) of exonic SSNVs shared between different regions of a primary tumor [42]. Several reports using aCGH and WGS approaches to analyze different PCa tumors also demonstrated this intra-tumoral heterogeneity [36, 43]. The report from Navin et al. further revealed a high-degree of heterogeneity at the cellular level by studying single cells from a dissociated tumor tissue [44]. The CTCs from our patient were obtained at a point in his natural history when he had both osseous and hepatic metastases. Overall, there should be a high degree of genomic heterogeneity at the intercellular, intra-tumoral, and inter-tumoral levels.

SVs, such as ETS rearrangements, constitute an important portion of founder mutations in PCa [45]. Acknowledging their importance in biology, we performed the first whole genome SV analysis on single CTCs. Our patient was not found to have an ETS fusion. However, we discovered two large segment rearrangement events shared between tumor tissues and CTCs (Figure 4). These SVs were undetected in WBCs and the normal tissue control. The presence of these shared rearrangements implies that they developed early in the course of disease evolution, which is consistent with the current understanding that some early rearrangements may be the drivers of PCa oncogenesis [28]. The SV and the SSNV analysis point toward a shared genetic origin for the CTCs and the metastatic lesion.

We also identified SVs involving important tumor suppressor genes, such as PTEN, in CTCs and tumor tissues (Figure 5). PTEN SVs are among the most common in PCa [46]. We observed extensive heterogeneity in both the CTCs and the tumor tissues. This observation suggested that these rearrangement events may have been acquired separately which is consistent with characterization of *PTEN* mutations as a late event in the evolution of PCa [47]. Before more powerful tools for SV detection are available, we point out that it may be difficult to identify which differences are meaningfully reflecting the heterogeneity of the disease. To address the growing interests in single-cell biology, new algorithms may be needed to identify SVs in single cells. We propose that that single-cell RNA sequencing may also be performed to cross validate the discovery of fusion products.

Similar to a previous study [20], we observed a trend (Figure 3A and 3C); the number of shared mutations discovered in the CTCs increased as we sequenced more CTCs. This would imply that at any given time point, multiple CTCs would be needed to better approximate the genomic content of the underlying disease (i.e. addressing spatial heterogeneity). Though a substantial amount of genomic information can be acquired from a limited number of CTCs, more CTCs would provide an even more comprehensive representation of the tumor genome. This is further complicated by the genetic evolution of the disease (i.e. temporal heterogeneity). To better address this issue, multiple CTCs would need to be captured at numerous time points. Also, Figure 4A shows that a large portion of shared mutations was found in CTCs as minor alleles with a small numbers of supporting reads. These mutations may have been underrepresented due to the preferential amplification of one allele during the WGA process. This suggests that increased sequencing depth may be needed to increase the sensitivity of the detection of mutations that present as minor alleles. In addition, circulating tumor DNA (ctDNA) may also provide an alternative source of materials for biological study [48–50]. Without the need of amplification, ctDNA sequencing may serve as a complementary strategy to reduce the false positive calls in single-CTC sequencing.

Prior published CTC sequencing efforts have resulted in lower coverage making WGS identification of CNVs, SVs, and SSNVs very challenging. Our methodology makes the analysis of CTC genomes with adequate quality for the assessment of SSNVs and SVs possible. Future work will focus on the utilization of CTC genomic characterizations from serially collected samples to perform a real-time, dynamic monitoring of a patient's cancer. Such a prospect is promising in diseases such as PCa, where tumor biopsies cannot be performed easily. Our single-CTC capture approach can also be modified to allow for the characterization of RNA instead of DNA. This will allow for the dynamic monitoring of the transcriptomes of metastatic castration-resistant PCa as well as in other cancers.

While the approach of performing multiple WGS studies on individual CTCs may seem impractical at this time, as sequencing technologies improve and as costs decline, this approach may be useful in understanding the dynamic evolution of heterogeneity in an underlying cancer. Genomic instability in cancer has been recognized as a potential barrier to personalized medicine in oncology given its relationship to therapeutic resistance. More recently, it has been hypothesized that this instability may result in larger generation of neoantigens increasing the benefit of immunotherapy [51, 52]. As such, this unique approach to dynamic characterization of heterogeneity may have clinical utility in the future.

In summary, our work demonstrates the feasibility of conducting WGS on single CTCs. This may provide a means to study cancer in real-time with simple serial phlebotomy as opposed to serial biopsy. Studies of this nature may provide valuable insights into the molecular mechanisms of disease progression.

## MATERIALS AND METHODS

### NanoVelcro chip for single CTCs isolation

We captured single CTCs for WGS using a NanoVelcro Chip composed of a nanofiber cell-affinity substrate and an overlaid chaotic mixer. This chip was used in conjunction with a laser capture microdissection (LCM) microscope to isolated the captured CTCs (Figure 1A and 1B) [18]. To ensure sufficient DNA input for WGS from the individually isolated CTCs, we performed WGA using multiple displacement amplification (MDA) on the DNA derived from single CTCs. A previous verified multiplex PCR of eight housekeeping genes was performed to assess the integrity of the amplified DNA (Figure 1C) [22]. Only high-quality WGA products were selected for library construction and WGS.

### Patient and samples

Collection of blood and tissue samples and clinical information in this study was approved by the Institutional Review Board at Cedars-Sinai Medical Center (CSMC). Written consent was obtained from the patient described herein. Archival FFPE tissues, including primary tumor tissue, adjacent normal and liver metastasis tissues were obtained from the pathology core at CSMC. Blood was also collected for CTC isolation over a span of 4 months.

We isolated single CTCs for WGS using a NanoVelcro Chip composed of 1) a cell-affinity substrate coated with poly(lactic-co-glycolic acid) (PLGA)-nanofibers and 2) an overlaid PDMS chaotic mixer. This chip was used in conjunction with a LCM microscope to isolated the captured CTCs [18]. In brief, PLGA-nanofibers were electrospun onto a LCM-compatible substrate. These nanofibers were covalently functionalized with streptavidin to enable selective capture of CTCs labeled with biotinylated anti-EpCAM. This surface facilitates CTC capture using its unique topographical interactions between the nanostructured components (e.g. microvilli) on the CTC surfaces and the PLGA-nanofibers on the NanoVelcro Chip. The PDMS chaotic mixer introduces helical flow that further increases the contact frequency between CTCs and the substrate surface. After CTCs immobilized on the substrate, the CTC-bearing substrate was stained with FITC-labeled anti-cytokeratin (CK), a standard marker for CTCs, and TRITC-labeled anti-CD45, a standard WBC marker. CTCs were defined as CK+/CD45- cells whose morphology could be confirmed using bright field microscopy. Finally, LCM was utilized to collect single CTCs. The WBCs in the flow-through of NanoVelcro CTC Chips were also collected.

### Single-CTC and tissue sequencing

Multiple displacement amplification was used to conduct WGA for individual CTCs. A multiplex PCR protocol was performed over 8 preselected housekeeping genes on different chromosomes for post-amplification quality assessment. Only samples with more than 6 housekeeping genes detected were considered high-quality and were utilized for WGS. DNA from tumor specimens from the archival tissues underwent the same amplification process. Non-tumor DNA was extracted from WBCs. WGS was performed on the Illumina HiSeq 2000 platform with paired-end 100 bp runs. WGS was carried out with paired-end 100-bp reads and ~350 bp inserts. Additional 8 CTCs with moderate quality were subjected to whole exome sequencing (WES). The DNA library was prepared using the TruSeq DNA kit (Illumina) followed by exome enrichment using the SeqCap EZ Human Exome library kit (v3.0, Roche).

We used the *Homo sapiens* reference genome sequence (hg19) and its annotation files (dbSNP v137) for WGS data analysis (Raw data available in GenBank, SRA121256). CNVs were assessed by CASS and FREEC [31, 32]. SSNVs were identified by MuTect 1.1.4 [25]. Founder SSNVs were identified as the SSNVs shared between the primary and metastatic tumors. Clonal SSNVs in the CTCs were defined as SNVs detected in at least 3 single CTCs [20, 53]. A modified Bayesian probabilistic model was utilized to assess the confidence of SSNV calls by considering ADO rates into the analysis. For the mutations repetitively observed in multiple WGS samples, algorithms including PolyPhen-2 were employed to predict their functional roles in disease progression [54]. Breakpoints of possible structural variations (SVs) were identified by CREST [27]. As CREST was not originally designed for our approach, supporting reads were verified manually for large segment rearrangements (see supplementary methods for detailed information).

## SUPPLEMENTARY DATA FIGURES AND TABLES








