# Supplementary material for: A comparison of isolated circulating tumor cells and tissue biopsies using whole-genome sequencing in prostate cancer
Source: Oncotarget. 2015 Nov 5;6(42):44781–93. doi: 10.18632/oncotarget.6330 (PMC4792591; doi:10.18632/oncotarget.6330)
Supplement: Supplementary file 1 [file oncotarget-06-44781-s001.pdf]

SUPPLEMENTARY DATA

DETAILED METHODS

The index patient and samples

A signed written consent was obtained before recruitment for the study, according to the regulations of the institutional review board at Cedars-Sinai Medical Center [CSMC] (IRB #3979). Fresh blood samples were collected from this patient in a 4 month span at Cedars-Sinai Medical Center. A liver biopsy of his metastatic tumor was obtained in the middle of the blood collection with FFPE preservation. His primary tumor and adjacent normal tissues 6 years prior to the study were obtained from the CSMC pathology archives.

CTC isolation by polymer nanofiber (PN)-nanovelcro CTC chips

We employed a previously described method for CTC isolation (1). Briefly, we fabricated our polymer nanofiber NanoVelcro substrate using an electrospinning method (2-4) for the deposition of PLGA nanofibers onto a commercially available laser micro-dissection (LMD) membrane slide. Then, we fabricated the PDMS chaotic mixer based on a "soft-lithography" approach (5), which utilizes a silicon mold with designed structures to identically replicate the structure of the PDMS chaotic mixer. After assembling the PDMS chaotic mixer onto our PN-NanoVelcro substrate, we create a microfluidic channel that facilitates the chemical modification and subsequent CTC capture on the PN-NanoVelcro CTC Chip.

To achieve specific immobilization of CTCs we utilized streptavidin to conjugate the polymer nanofibers to facilitate the capture of CTCs labeled with biotinylated anti-EPCM. Briefly, 0.5 mL EDC (8 mg mL^{-1}) and sulfo-NHS (2 mg mL^{-1}) is prepared with 1x PBS and is slowly loaded into the channel to convert the carboxyl group on the terminal of the PLGA molecule to an amine-reactive sulfo-NHS ester. The channel is then rinsed multiple times with a PBS solution to eliminate the free EDC and sulfo-NHS. Streptavidin (250 μg mL^{-1}) is then introduced into the channel, which reacts with the sulfo-NHS ester. After removal of the free streptavidin molecules, the PN-NanoVelcro chip coated with streptavidin is then rinsed with PBS multiple times and is ready for subsequent blood processing.

Patient blood samples were collected into EDTA-containing vacutainer tubes (BD bioscience) according to standard phlebotomy protocols. Whole blood was treated with an RBC lysis buffer (Biolegend) following the manufacturer's protocol. The peripheral blood mononuclear cells (PBMCs) were treated with biotinylated anti-EpCAM (R&D, 8μg mL-1, in 200μL PBS with 2% (w/v) BSA) at room temperature for 0.5h. After washing, the PBMCs (200μL) along with the biotinylated anti-EpCAM labeled CTCs were then loaded into 1 mL disposable syringes and introduced into the PN-NanoVelcro CTC Chips at a constant flow rate (0.5 mL/hr) via a syringe pump (KDS200, KD-Scientific). After processing, captured cells were fixed by 100% ice-cold ethanol. After rinsing away the remaining ethanol, an antibody cocktail consisting of FITC-conjugated anti-CK and PE-conjugated anti-CD45 (in 2% Donkey Serum) was used for immunocytochemistry staining for one hour at room temperature away from light. After staining, the PN-NanoVelcro substrate was first washed by PBS and then dissembled from the PDMS chaotic mixer. After drying for 10 minutes in a vacuum container, the chips were loaded into a LCM microscope (ArcturusXT™, Life Technologies) for CTC isolation.

In order to achieve the isolation of high purity single-CTCs, CTCs were first identified under the LCM microscope based on their morphology and fluorescence staining (CK+/CD45-). Selective dissection of CTCs was achieved by UV laser, and the dissected single CTCs were captured by the IR-activated conical polymer pillar onto a CapSure™ HS Cap. Finally, the HS Caps with dissected CTCs were stored in -80°C until analysis could be performed. To ensure these CTCs are free of contamination, the entire procedure is carried out in a Class 1000 clean room in the core facility of the California NanoSystems Institute at UCLA.

Single-CTC and tissue sample preparation and sequencing

After obtaining single CTCs, WGA is achieved on these samples using the REPLI-g Single Cell Kit according to the manufacturer's manual (QIAGEN GmbH). A reaction of a total volume of 50 ml was performed at 30°C for 8 hours and then terminated at 65°C for 10 min. Amplified DNA products were stored at -20°C. For quality assessment of the amplification product, a multiplex PCR protocol was performed over eight preselected housekeeping genes on different chromosomes, with the assumption that the integrity of these areas may represent the amplification quality of the entire genome. In our protocol, single-cell WGA products with successful PCR

products in more than 6 housekeeping genes were chosen as qualified samples for subsequent sequencing.

For the FFPE archival primary tumor tissue, adjacent normal and liver metastasis tissues, the tissue core biopsies were obtained from the pathology core at CSMC. Tumor content in the primary tumor was >80%, and the tumor content was close to 100% in the metastatic liver tumor. DNA extraction was performed on these samples using the RecoverAll™ Total Nucleic Acid Isolation Kit for FFPE (Life Technologies). For the metastatic liver biopsy, due to the insufficient quantity of the FFPE DNA, we performed WGA by the REPLI-g FFPE Kit (Qiagen), which consists of random ligation of DNA fragments and MDA. For normal WBC control samples, we collected the flow-through from the PN-NanoVelcro CTC Chips, which are void of CTCs. This flow-through went through DNA extraction by the DNeasy tissue and blood kit (Qiagen). Due to unsatisfactory DNA quantity, direct MDA was performed using the REPLI-g Single Cell Kit.

DNA libraries were prepared using the TruSeq DNA kit (Illumina). The DNA library was then put on the Illumina CBot for template enrichment. Sequencing was performed on the Illumina HiSeq 2000 platform with paired-end 100 bp runs. For the WBC, tumor tissue and CTC samples, the targeted sequencing depths were 30X. For normal adjacent tissue, due to the insufficient DNA material, the targeted sequencing depth was 10X.

WGS data analysis

We used the Human (Homo sapiens) reference genome sequence (hg19) and its annotation files (dbSNP v137) for our analysis. They were downloaded from the University of California Santa Cruz Genome Bioinformatics website (http://genome.ucsc.edu/). For the WGS analysis pipeline, BWA 0.6.2 (6) was used for the alignment. After removing PCR duplicates by Picard 1.72, GATK 2.2.3 was used for indel-realignment and base quality recalibration (7). SNPs were called by the GATK unified genotyper algorithm.

Lorenz curves were used to assess the coverage uniformity in the single-CTC WGS data. Briefly, 100 million reads were randomly sampled from the WGS data. The human genome reference (hg19) was equally divided into 100 segments and the number of reads aligned to each area was calculated. The cumulative fraction of the total reads that cover a given cumulative fraction of the genome was used to calculate the Gini coefficient using ineq package in R. The Lorenz curve of our single-CTC WGS was compared with previously published single-cell sequencing data (8). The diagonal line indicates a perfectly uniform distribution of reads, and deviation from the diagonal line indicates an uneven distribution of reads. Raw data will be available in GenBank with the Accession number SRA121256.

SSNV analysis

SSNVs were identified by the following methods:

1. The bam file of a tumor genome was directly compared with the WBC reference using MuTect 1.1.4 (9).
2. These SSNVs were not present in the dbSNP 137 database.
3. The SSNVs in the normal adjacent tissue was identified by the comparison of normal tissue to the WBC reference via the MuTect algorithm. The SSNVs identified from the differences between normal adjacent tissue and WBC were used to further filter the SSNVs of CTCs and tumor tissues.

Founder SSNVs were identified as the SSNVs shared between the primary and metastatic tumors. Clonal SSNVs in the CTCs were defined as SNVs detected in at least three single CTCs. For the assessment of supporting reads of founder SSNVs in the CTC WGS, Samtools mpileup commend was used to examine the presence of reads supporting the founder SSNVs in the CTCs. The Samtools mpileup commend was also used to examine the primary and metastatic tumors for the presence of supporting reads of the clonal SSNVs in CTCs.

Statistical model for assessing the probability of SSNVs in single-CTCs

Bayesian probabilistic models are widely used in common SSNV calling tools, such as GATK for calling SNPs and Mutect for calling SSNVs. However, for single-cell sequencing data, we have to take into account the ADO rate when assessing the confidence of SSNV calls. For each site, denote *bi* and *ei* as the called base and error probability of the *i*th read ($i = 1 \ldots n$) spanning the site from a single-CTC sequencing data. We then calculate the posterior probability for the true diploid genotype *Gk* in { AA, AC, AG, AT, CC, CG, CT, GG, GT, TT } of the single-CTC given the sequencing data by averaging out all possible sequenced genotypes *Al* that might have an allele dropped out after WGA:

$$P(G_k|\{b_i\},\{e_i\}) = \frac{\sum_{l=0}^{9} P(G_k)P(A_l|G_k)P(\{b_i\}|A_l,\{e_i\})}{\sum_{j=0}^{9}\sum_{l=0}^{9} P(G_j)P(A_l|G_j)P(\{b_i\}|A_l,\{e_i\})}.$$

Where $P(\{b_i\}|A_l,\{e_i\}) = \Pi_{i=1}^{n} p(b_i|A_i,e_i)$ is the sequenced genotype likelihood, such that

$$P(b_i|A_l,e_i) = \begin{cases} \frac{1}{2}(1-e_i) + \frac{1}{2}(1-e_i) & \text{if } b_i = A_{l1} = A_{l2} \\ \frac{1}{2}(1-e_i) + \frac{1}{2}(\frac{e_i}{3}) & \text{if } b_i = A_{l1} \neq A_{l2} \text{ or } b_i = A_{l2} \neq A_{l1} \\ \frac{1}{2}(\frac{e_i}{3}) + \frac{1}{2}(\frac{e_i}{3}) & \text{otherwise} \end{cases}$$

and $P(A_l|G_k)$ is the probability of sequenced genotype given the true genotype, such that

$$P(A_l|G_k) = \begin{cases} 1 - e_{ADO} & if\, A_l = G_k \\ \frac{1}{2}e_{ADO} & if\, A_l = G_{k1}G_{k1}\, or\, G_{k2}G_{k2} \\ 0 & otherwise \end{cases}$$

where e_{ADO} is the ADO rate and we assume the two alleles have the same chance to be dropped out. For the prior probability, we use the equation published by Larson et al(10)., such that

$$P(G_k) = \begin{cases} \pi & if\, R = G_{k1} \neq G_{k2}\, or\, R = G_{k2} \neq G_{k1} \\ \pi^2 & if\, R \neq G_{k1}\, and\, R \neq G_{k2} \\ 1 - \sum_{G_j \neq RR} P(G_j) & otherwise \end{cases}$$

where R is the reference base and $\pi = 10^{-5}$is the expected rate of heterozygous mutations in human samples. For a specific alternative allele M, the LOD score is defined as

$$LOD = log_{10}\frac{P(RM|\{b_i\}, \{e_i\}) + P(MM|\{b_i\}, \{e_i\})}{P(RR|\{b_i\}, \{e_i\})}.$$

For the determination of ADO, since there is no CTC bulk sequencing that can be compared with single-CTC data, we utilized an estimation approach. First, the heterozygous loci in the WBC, primary and metastatic tumors were determined individually by the GATK unified genotyper. Due to the possible loss of heterozygosity in tumor cells, we selected only loci present as heterozygous in all three of the tissues into our analysis. The genotype of each single-CTC was compared with the abovementioned list of loci. The ADO was estimated as the total number of homozygous loci in the list divided by the total number of loci.

A heatmap was constructed using the LOD score. For the concern of visualization, we truncated the maximum LOD score to 1, where LOD≥1 means the posterior probability of the CTC harboring the specific alternative allele is greater than 10 times the probability of no mutations. We note the minimum value -5 was introduced by the prior probability of observing the somatic mutation. Thus, LOD≥-5 means that if we ignore the prior probability, the likelihood of the CTC harboring the specific alternative allele is greater than no mutations.

WES of CTCs

CTCs isolated for WGA that showed one to five bands with correct product size from the multiplex PCR were marked as suboptimal for WGS. Eight of these CTCs isolated from samples collected from July to September 2012 were sent for WES. The DNA library was prepared using the TruSeq DNA kit (Illumina) followed by exome enrichment using the SeqCap EZ Human Exome library kit (v3.0, Roche). Enriched exome DNA was then put on the Illumina CBot for template enrichment. Sequencing was performed on the Illumina HiSeq2000 platform with paired-end 100 bp runs. The SSNVs in the CTC WES were analyzed utilizing exactly the same pipeline described above.

Validation of variations

Due to the fact that false positives can be introduced during the amplification process of single-cell DNA, validation of our findings couldn't be performed on the same cells. Therefore, our mutation sites were validated by the following approach. First, we performed additional whole exome sequencing to validate that the somatic exonic mutations sites were not present in WBCs. In brief, DNA from another batch of WBCs was extracted using the same method. The DNA library was then prepared using the TruSeq DNA kit (Illumina) followed by exome enrichment using the SeqCap EZ Human Exome library (v3.0, Roche). Enriched exome DNA was then put on the Illumina CBot for template enrichment. Sequencing was performed on the Illumina HiSeq2000 platform with paired-end 100 bp runs. The data alignment was performed as mentioned earlier, but the genotypes of all bases were called by GATK. For the validation of false somatic mutations, randomly selected somatic mutation sites were checked in this new dataset. The success of validation was defined as somatic mutation sites with REF genotypes found in the WBC exome sequencing.

Randomly selected SSNVs were further validated by Sanger sequencing. A total of 30 exonic mutations were selected for validation in all tumor and WBC samples. Among them, five of them did not have suitable primers for PCR amplification. The success of validation is defined as the detection of identified mutations in any of the tumor tissues divided by the total number of sites with PCR products.

Structural variation analysis

For the analysis of structural variations (SVs), the CREST algorithm (14) was used for the identification of breakpoints of possible SVs. Due to the concern that CREST was not designed for FFPE and WGA tissues, the identification of supporting reads of the SVs were done manually on large segment rearrangements. In brief, the paired-end reads near the two ends of the identified breakpoints were plotted. The reads with indels were eliminated to avoid possible mapping errors. The reads with two ends on each sides of the breakpoint were identified as supporting reads. The supporting reads condition was also assessed using WBC and normal adjacent tissue controls to validate the somatic nature of the SVs. For the

SVs in the important oncogenes, the lengths of these SVs are mostly < 1kb, so that they cannot be assessed by the manual validations of supporting reads. For the validation of ETS rearrangement status, reads in ERG and its reported fusion partners, SLC45A3, HERPUD1, TMPRSS2 and NDRG1 were examined. SVs in PTEN, RB1 and BRCA2 were too small to be validated by the supporting reads method.

CNV analysis

CASS (15) was used for the identification of CNV analysis with a floating window of 100kb in size. The identified CNVs were filtered based on their p-values (<0.05). FREEC(16) with the setting of a 100kb fixed window was used to compare the tumor and normal tissue for the construction of Figure 2D and S5. The final results of SSNV analysis were based on the overlap areas between those identified by CASS and FREEC. For the aCGH, the DNA from the FFPE preserved primary prostate tumor tissue was prepared using the Recoverall total nucleic acid isolation kit (Ambion, Life Technologies). The CytoScan HD array assay (Affymetrix) was performed following the manufacturer's manual. The results were analyzed using the Affymetrix Chromosome Analysis Suite (ChAS) 2.0 software.

REFERENCES

1. Zhao L, Lu YT, Li F, Wu K, Hou S, Yu J, et al. High-Purity Prostate Circulating Tumor Cell Isolation by a Polymer Nanofiber-Embedded Microchip for Whole Exome Sequencing. Adv Mater 2013.
2. Shin HJ, Lee CH, Cho IH, Kim YJ, Lee YJ, Kim IA, et al. Electrospun PLGA nanofiber scaffolds for articular cartilage reconstruction: mechanical stability, degradation and cellular responses under mechanical stimulation in vitro. J Biomater Sci Polym Ed 2006; 17:103–19.
3. Xin X, Hussain M, Mao JJ. Continuing differentiation of human mesenchymal stem cells and induced chondrogenic and osteogenic lineages in electrospun PLGA nanofiber scaffold. Biomaterials 2007; 28:316–25.
4. Kim SJ, Jang DH, Park WH, Min BM. Fabrication and characterization of 3-dimensional PLGA nanofiber/microfiber composite scaffolds. Polymer 2010; 51:1320–27.
5. Wang ST, Liu K, Liu JA, Yu ZTF, Xu XW, Zhao LB, et al. Highly Efficient Capture of Circulating Tumor Cells by Using Nanostructured Silicon Substrates with Integrated Chaotic Micromixers. Angew Chem Int Edit 2011; 50:3084–88.
6. Li H, Durbin R. Fast and accurate short read alignment with Burrows-Wheeler transform. Bioinformatics 2009; 25:1754–60.
7. DePristo MA, Banks E, Poplin R, Garimella KV, Maguire JR, Hartl C, et al. A framework for variation discovery and genotyping using next-generation DNA sequencing data. Nature genetics 2011; 43:491–8.
8. Zong C, Lu S, Chapman AR, Xie XS. Genome-wide detection of single-nucleotide and copy-number variations of a single human cell. Science 2012;338:1622–6.
9. Cibulskis K, Lawrence MS, Carter SL, Sivachenko A, Jaffe D, Sougnez C, et al. Sensitive detection of somatic point mutations in impure and heterogeneous cancer samples. Nature biotechnology 2013; 31:213–9.
10. Larson DE, Harris CC, Chen K, Koboldt DC, Abbott TE, Dooling DJ, et al. SomaticSniper: identification of somatic point mutations in whole genome sequencing data. Bioinformatics 2012; 28:311–7.
11. Kumar P, Henikoff S, Ng PC. Predicting the effects of coding non-synonymous variants on protein function using the SIFT algorithm. Nature protocols 2009; 4:1073–81.
12. Choi Y, Sims GE, Murphy S, Miller JR, Chan AP. Predicting the functional effect of amino acid substitutions and indels. PloS one 2012; 7:e46688.
13. Adzhubei IA, Schmidt S, Peshkin L, Ramensky VE, Gerasimova A, Bork P, et al. A method and server for predicting damaging missense mutations. Nature methods 2010; 7:248–9.
14. Wang J, Mullighan CG, Easton J, Roberts S, Heatley SL, Ma J, et al. CREST maps somatic structural variation in cancer genomes with base-pair resolution. Nature methods 2011; 8:652–4.
15. Zhang C, Chen S, Yin X, Pan X, Lin G, Tan Y, et al. A single cell level based method for copy number variation analysis by low coverage massively parallel sequencing. PloS one 2013; 8:e54236.
16. Boeva V, Popova T, Bleakley K, Chiche P, Cappo J, Schleiermacher G, et al. Control-FREEC: a tool for assessing copy number and allelic content using next-generation sequencing data. Bioinformatics 2012; 28:423–5.
17. Jariwala U, Prescott J, Jia L, Barski A, Pregizer S, Cogan JP, et al. Identification of novel androgen receptor target genes in prostate cancer. Molecular cancer 2007; 6:39.
18. Zhou Q, Anderson DJ. The bHLH transcription factors OLIG2 and OLIG1 couple neuronal and glial subtype specification. Cell 2002; 109:61–73.
19. Lin D, Wyatt AW, Xue H, Wang Y, Dong X, Haegert A, et al. High fidelity patient-derived xenografts for accelerating prostate cancer discovery and drug development. Cancer Res 2014; 74:1272–83.
20. Singh K, Sinha S, Malonia SK, Bist P, Tergaonkar V, Chattopadhyay S. Tumor suppressor SMAR1 represses IkappaBalpha expression and inhibits p65 transactivation through matrix attachment regions. The Journal of biological chemistry 2009; 284:1267–78.
21. Mancardi DA, Albanesi M, Jonsson F, Iannascoli B, Van Rooijen N, Kang X, et al. The high-affinity human IgG receptor FcgammaRI (CD64) promotes IgG-mediated

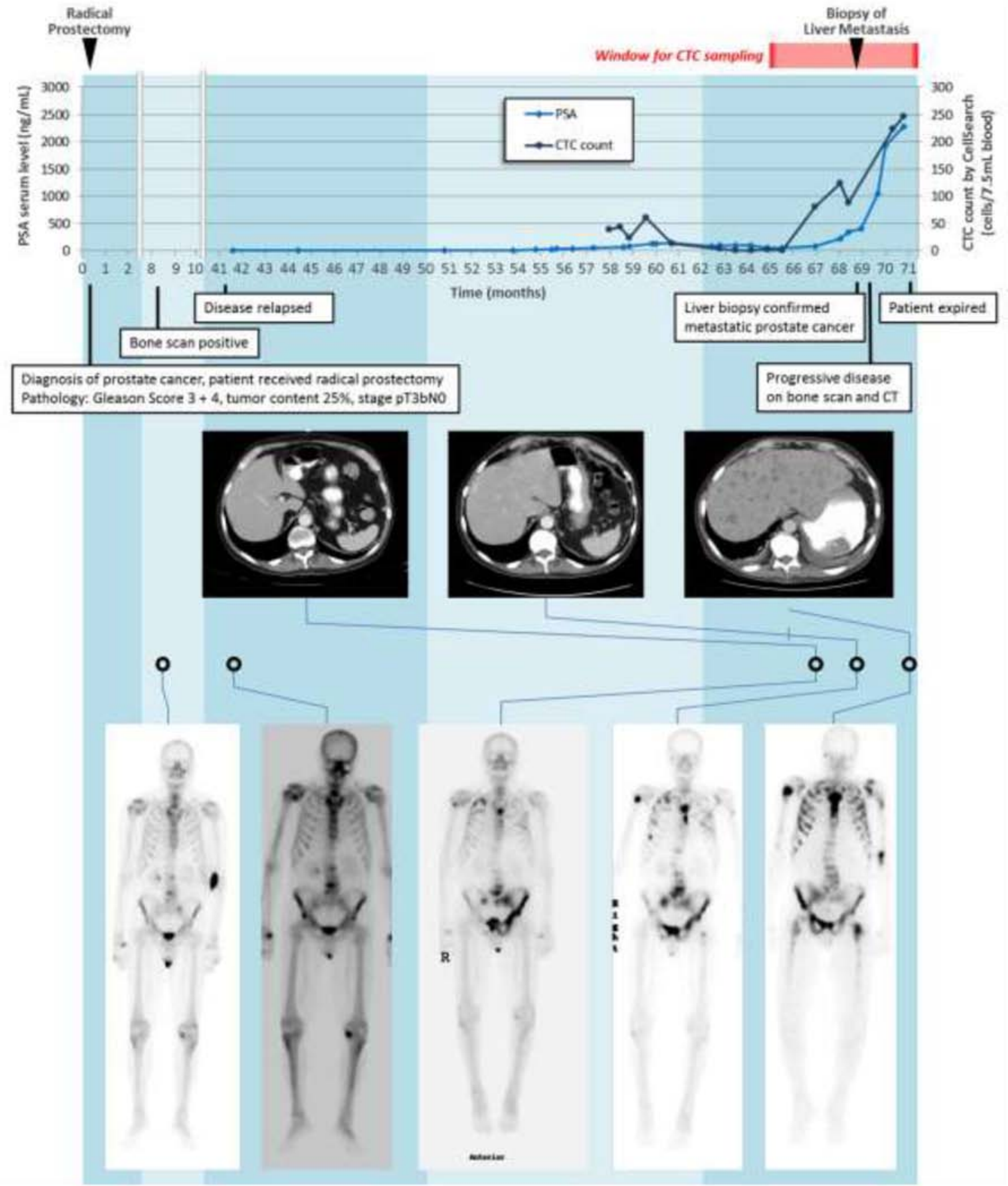

Supplementary Figure S1: Treatment course of our index patient and sample collection times.

inflammation, anaphylaxis, and antitumor immunotherapy.
Blood 2013;121:1563–73.

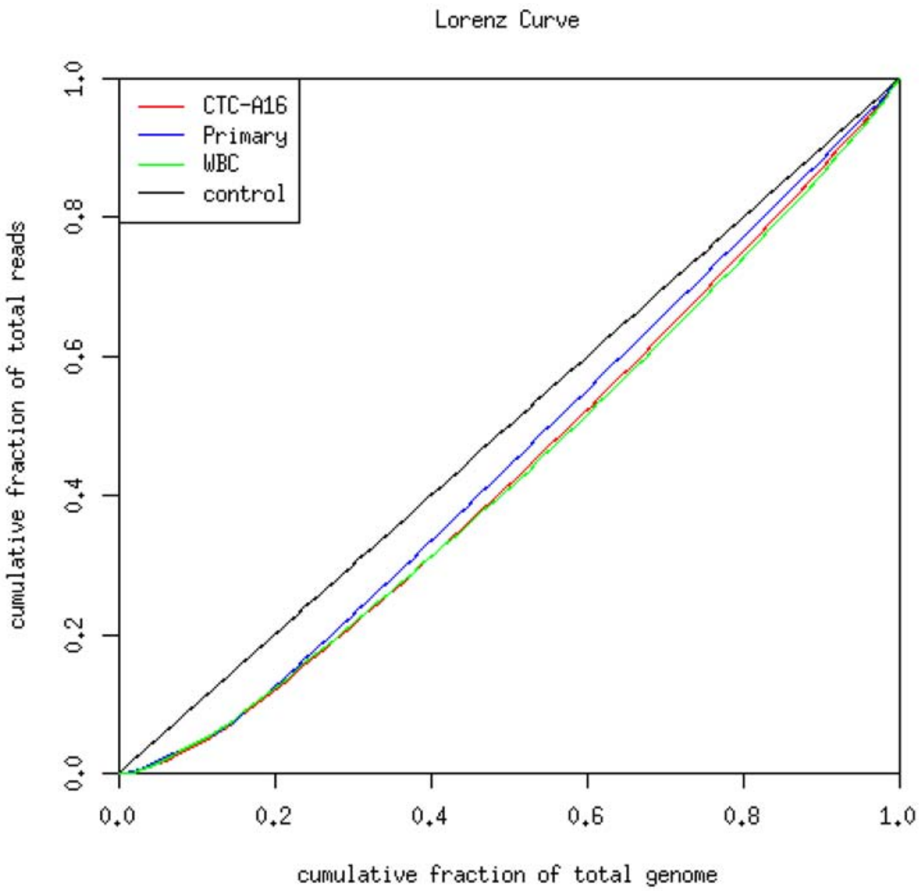

Supplementary Figure S2: Lorenz curves of CTC, primary tumor and WBC.

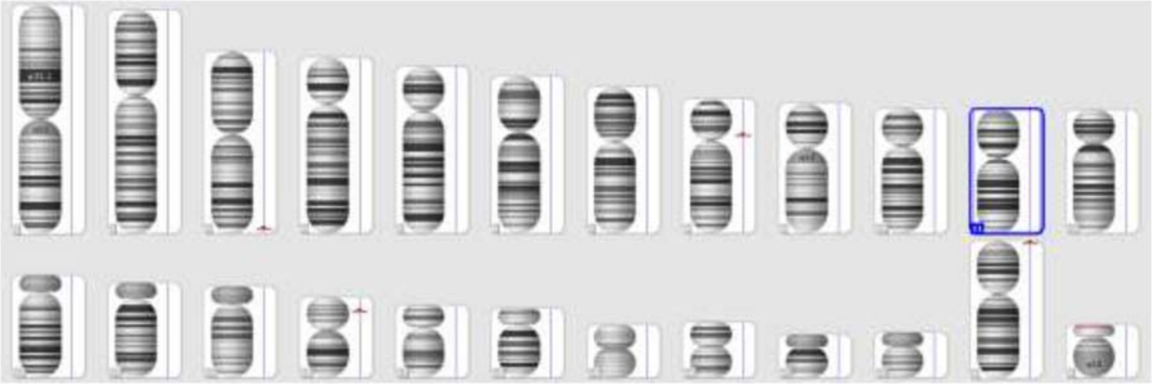

Supplementary Figure S3: aCGH confirmation of CNV in primary tumor. Only four areas of deletion were detected in the array. Their positions were chr16: 14928345-15129892, chr8: 39254032-39386952 (near centromere), chrX: 1656710-1779326 (near end) and chr3: 193286181-193404536 (near end). None of them were found in our WGS data.

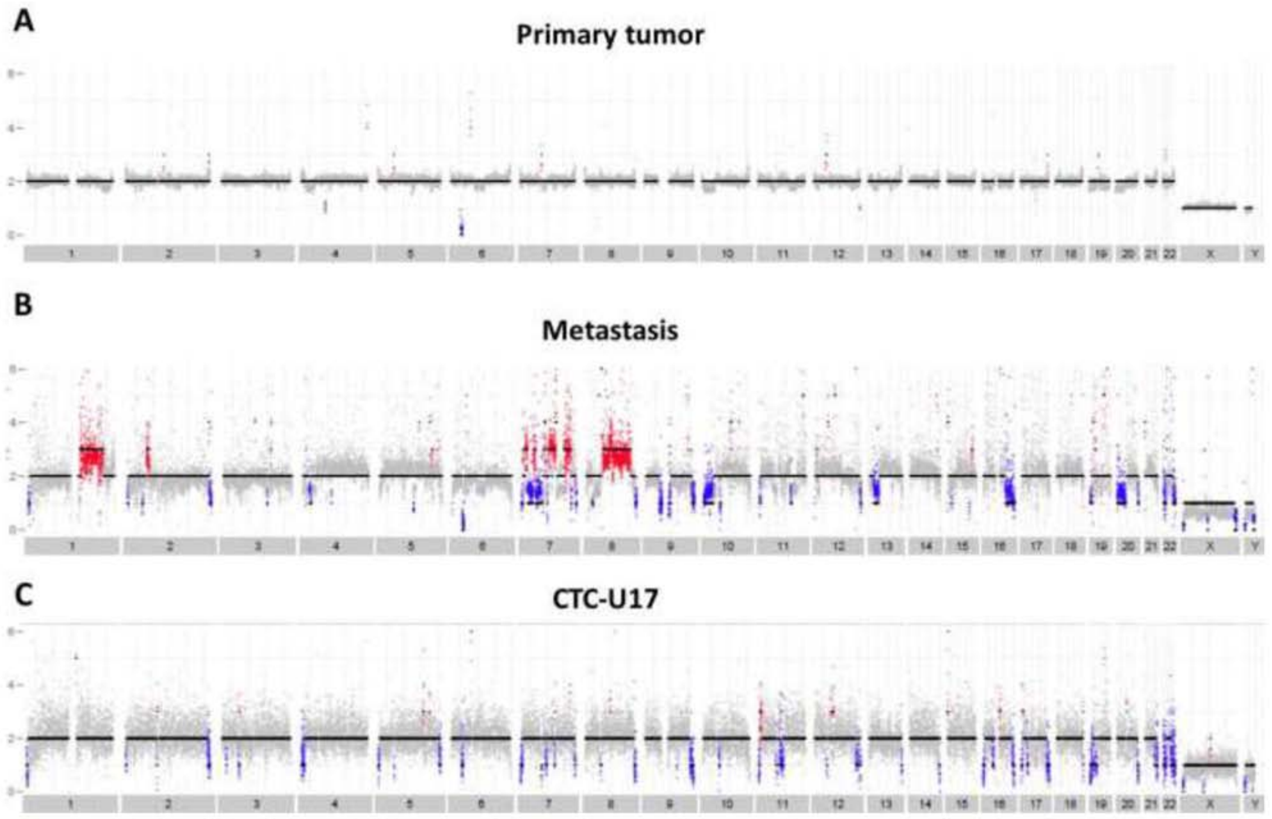

Supplementary Figure S4: CNV of primary tumor, metastasis and CTC.

Supplementary Table S1: WGS sequencing quality

Sample ID	All Reads (number)	All Mapped Reads (number)	All Mapped Base (bp)	All Depth (x)	All Coverage (%)	Uniquely Mapped Reads (number)	Uniquely Mapped Base (bp)	Unique Depth (x)	Unique Coverage (%)
CTC-A16	1,044,100,108	994,225,396	96,485,069,430	33.72	99.26	949,875,202	92,164,093,715	32.21	97.35
CTC-A9	1,073,912,790	1,020,037,130	98,685,211,851	34.49	95.57	973,593,850	94,165,657,855	32.91	93.65
CTC-U15	1,099,416,824	1,052,119,642	102,048,261,187	35.66	99.09	1,005,164,800	97,460,191,373	34.06	97.21
CTC-U17	1,097,954,942	1,056,888,532	103,144,513,739	36.05	99.55	1,010,312,202	98,573,089,192	34.45	97.66
Primary	1,043,123,234	986,159,338	89,563,157,396	31.3	99.89	925,694,490	83,670,062,824	29.24	98.29
Metastasis	1,122,272,220	1,035,155,834	80,244,016,628	28.04	99.67	986,300,687	75,561,613,161	26.41	98.53
WBC	1,084,756,410	1,034,068,020	99,615,402,559	34.81	99.72	989,004,520	95,196,502,949	33.27	97.79
Normal tissue	344,981,326	321,932,550	24,446,982,645	8.54	98.09	309,527,040	23,218,409,944	8.11	95.63

Supplementary Table S2: GC content correlation between single-CTC WGS and tissue WGS

Supplementary Table S3: SNP and indel analysis of WGS

Supplementary Table S4: Validation of SSNV sites
