# Supplementary material for: A comparison of isolated circulating tumor cells and tissue biopsies using whole-genome sequencing in prostate cancer
Source: Oncotarget. 2015 Nov 5;6(42):44781–93. doi: 10.18632/oncotarget.6330 (PMC4792591; doi:10.18632/oncotarget.6330)
Supplement: Supplementary file 2 [file oncotarget-06-44781-s002.docx]

| **Table S2** GC content correlation between single-CTC WGS and tissue WGS | | | | | | | | |
| --- | --- | --- | --- | --- | --- | --- | --- | --- |
|  | CTC-A16 | CTC-A9 | CTC-U15 | CTC-U17 | WBC | Normal tissue | Metastasis | Primary |
| CTC-A16 | 1.000000000000000 | 1.000000000000000 | 1.000000000000000 | 1.000000000000000 | 0.999999999999163 | 0.999999999999163 | 1.000000000000000 | 1.000000000000000 |
| CTC-A9 | 1.000000000000000 | 1.000000000000000 | 1.000000000000000 | 1.000000000000000 | 0.999999999999163 | 0.999999999999163 | 1.000000000000000 | 1.000000000000000 |
| CTC-U15 | 1.000000000000000 | 1.000000000000000 | 1.000000000000000 | 1.000000000000000 | 0.999999999999163 | 0.999999999999163 | 1.000000000000000 | 1.000000000000000 |
| CTC-U17 | 1.000000000000000 | 1.000000000000000 | 1.000000000000000 | 1.000000000000000 | 0.999999999999163 | 0.999999999999163 | 1.000000000000000 | 1.000000000000000 |
| WBC | 0.999999999999163 | 0.999999999999163 | 0.999999999999163 | 0.999999999999163 | 1.000000000000000 | 1.000000000000000 | 0.999999999999163 | 0.999999999999163 |
| Normal tissue | 0.999999999999163 | 0.999999999999163 | 0.999999999999163 | 0.999999999999163 | 1.000000000000000 | 1.000000000000000 | 0.999999999999163 | 0.999999999999163 |
| Metastasis | 1.000000000000000 | 1.000000000000000 | 1.000000000000000 | 1.000000000000000 | 0.999999999999163 | 0.999999999999163 | 1.000000000000000 | 1.000000000000000 |
| Primary | 1.000000000000000 | 1.000000000000000 | 1.000000000000000 | 1.000000000000000 | 0.999999999999163 | 0.999999999999163 | 1.000000000000000 | 1.000000000000000 |
