# Supplementary material for: A comparison of isolated circulating tumor cells and tissue biopsies using whole-genome sequencing in prostate cancer
Source: Oncotarget. 2015 Nov 5;6(42):44781–93. doi: 10.18632/oncotarget.6330 (PMC4792591; doi:10.18632/oncotarget.6330)
Supplement: Supplementary file 3 [file oncotarget-06-44781-s003.docx]

| **Table S3** SNP and indel analysis of WGS | | | | | | | | | | | | | |
| --- | --- | --- | --- | --- | --- | --- | --- | --- | --- | --- | --- | --- | --- |
| Sample ID | SNP Number | Noval Rate | Heterozygosis Rate | Missense Rate | Nonsense Rate | Silent Rate | Transitions/Transversions Rate | A>C T>G | A>G T>C | A>T T>A | C>G G>C | C>T G>A | G>T C>A |
| CTC-A16 | 3,358,240 | 5.14% | 53.96% | 46.48% | 0.48% | 53.04% | 2.0727 | 273,147 | 1,074,945 | 242,827 | 281,961 | 1,190,361 | 294,999 |
| CTC-A9 | 2,756,806 | 7.32% | 39.84% | 48.64% | 0.93% | 50.43% | 1.9737 | 223,941 | 878,380 | 202,681 | 229,773 | 951,354 | 270,677 |
| CTC-U15 | 3,274,148 | 4.22% | 52.37% | 46.38% | 0.43% | 53.18% | 2.0354 | 269,371 | 1,058,565 | 238,381 | 278,853 | 1,136,911 | 292,067 |
| CTC-U17 | 3,614,078 | 2.97% | 60.03% | 45.36% | 0.27% | 54.37% | 2.0305 | 299,892 | 1,176,280 | 266,197 | 308,517 | 1,245,211 | 317,981 |
| Primary | 3,557,384 | 3.13% | 60.15% | 44.87% | 0.21% | 54.93% | 2.0166 | 295,178 | 1,160,671 | 263,978 | 305,944 | 1,217,436 | 314,177 |
| Metastasis | 3,230,010 | 6.37% | 57.30% | 45.55% | 0.27% | 54.19% | 2.0182 | 269,028 | 1,057,352 | 243,730 | 276,041 | 1,102,497 | 281,362 |
| WBC | 3,569,526 | 4.09% | 58.56% | 45.94% | 0.81% | 53.25% | 1.9619 | 293,110 | 1,151,822 | 259,104 | 302,899 | 1,212,553 | 350,038 |
| Normal tissue | 2,325,565 | 1.27% | 47.95% | 45.93% | 0.20% | 53.87% | 2.0204 | 200,007 | 772,033 | 175,680 | 195,245 | 783,591 | 199,009 |
|  |  |  |  |  |  |  |  |  |  |  |  |  |  |
|  |  |  |  |  |  |  |  |  |  |  |  |  |  |
| Sample ID | Indel Number | Noval Rate | Insert Number | Deletion Number | Heterozygosis Rate |  |  |  |  |  |  |  |  |
| CTC-A16 | 448,492 | 12.85% | 216,373 | 232,119 | 49.92% |  |  |  |  |  |  |  |  |
| CTC-A9 | 358,993 | 16.67% | 178,648 | 180,345 | 41.54% |  |  |  |  |  |  |  |  |
| CTC-U15 | 443,229 | 13.15% | 213,712 | 229,517 | 49.66% |  |  |  |  |  |  |  |  |
| CTC-U17 | 522,287 | 12.28% | 247,852 | 274,435 | 56.46% |  |  |  |  |  |  |  |  |
| Primary | 474,670 | 10.69% | 225,391 | 249,279 | 53.70% |  |  |  |  |  |  |  |  |
| Metastasis | 359,780 | 9.00% | 171,316 | 188,464 | 50.15% |  |  |  |  |  |  |  |  |
| WBC | 524,975 | 13.43% | 251,969 | 273,006 | 54.36% |  |  |  |  |  |  |  |  |
| Normal tissue | 163,097 | 6.09% | 78,219 | 84,878 | 36.95% |  |  |  |  |  |  |  |  |
