# Supplementary material for: A comparison of isolated circulating tumor cells and tissue biopsies using whole-genome sequencing in prostate cancer
Source: Oncotarget. 2015 Nov 5;6(42):44781–93. doi: 10.18632/oncotarget.6330 (PMC4792591; doi:10.18632/oncotarget.6330)
Supplement: Supplementary file 4 [file oncotarget-06-44781-s004.docx]

| **Table S4** Validation of SSNV sites | | | | | | | | | |
| --- | --- | --- | --- | --- | --- | --- | --- | --- | --- |
| Chr | Mutation Position | Faorward Primer Start Position | Reverse Primer Start Position | Product Size | Forward Primer | Reverse Primer | Validation Results | WGS Samples Carrying  the Mutation | WGS Samples Carrying the  Validated Mutation |
| chr1 | 16349121 | 16348913 | 16349225 | 294 | ATGACACACACAGGCACACA | CTGGCTCTCACCTTGGATGG | No | 1139-U15 | No Product |
| chr1 | 32658015 | 32657885 | 32658287 | 384 | CGACTGCTCCCACCATCTTT | CCTCTGGGACTCTACTGCCT | TRUE | 1139-A16 | 1139-A16 |
| chr1 | 149761661 | 149761510 | 149761689 | 161 | TCTAGGGCCAGGTTTCCCAA | TTCACAGCTCAGGGTGACCA | TRUE | 1139-A16,1139-U15 | 1139-A16,1139-U15 |
| chr1 | 181767882 | 181767642 | 181767981 | 321 | CTTGCCTGACCGAGTCTTCC | GGGAGCAGCCTCTAGCATTT | TRUE | 1139-A16 | 1139-A16 |
| chr1 | 183514104 | 183513589 | 183514298 | 690 | CATTCATCACCCTGGAGCCTT | GGGAAGGTGGCTGTGATTGT | TRUE | 1139-A9 | 1139-A9 |
| chr1 | 200842856 | 200842822 | 200843091 | 251 | CTTCTGCTACTGCCGCATCT | AGGAGGTAGATGAGCGGGTT | TRUE | UCLA-MET,UCLA-PCA | UCLA-MET |
| chr1 | 205628613 | 205628416 | 205628636 | 202 | GTAAATGGCGACCAGACCCA | CCTCTGCCTGTGATGTCTCC | FALSE | UCLA-MET | FALSE |
| chr1 | 231403565 | 231403292 | 231403591 | 281 | TGTTTGCACCTGACCTGAGA | TTCCACCAGAGCATCAAAGTCC | TRUE | 1139-A9 | 1139-A9 |
| chr10 | 38344566 | 38344396 | 38344724 | 310 | ATCCTTCCGTGTGACTTCGC | ATTCGGGACATGCAAAGGGT | TRUE | 1139-U15 | 1139-U15 |
| chr10 | 70940144 | 70940052 | 70940622 | 552 | CCTTCTCCCGTGCCCTATTG | GGCGTCCACCTAACAACTCA | TRUE | 1139-A16 | 1139-A16 |
| chr10 | 98405339 | 98405189 | 98405368 | 161 | TCAGCAGAAAGGTGGAAGCA | GAGGAGGCTGATGCTGTGTA | No | 1139-A16 | No Product |
| chr10 | 102748787 | 102748768 | 102749048 | 263 | TGACAGCCTGGCCTTGAAC | GCAGGGCGGTACGAAGAATA | TRUE | 1139-U17 | 1139-U17 |
| chr10 | 115947782 | 115947326 | 115948026 | 682 | GGAATGGTGTTGAACGCTGA | AGTCATGTGCCTGTTCTGCT | TRUE | UCLA-MET,UCLA-PCA | UCLA-MET,UCLA-PCA |
| chr10 | 126454068 | 126453257 | 126454098 | 823 | ATGACCGCTAGTGCAAGACA | GCCTTAATCCTGACAATGCAA | No | 1139-A9 | No Product |
| chr10 | 126660637 | 126660532 | 126660887 | 337 | AGGTGTCTCAACAAGCAGCA | CCTCTCAGATAGCAGACGCAT | TRUE | 1139-A9 | 1139-A9 |
| chr11 | 33564953 | 33564626 | 33565091 | 447 | CTTTCCTCCAGCCCACAGAG | AACCCAGAATGGTCCACAGC | TRUE | 1139-A9 | 1139-A9 |
| chr11 | 77916948 | 77916858 | 77916978 | 102 | ATCCTGCCTGCCTGCTTATT | GATAGAAACCCGCCAGCTC | TRUE | 1139-U17 | 1139-U17 |
| chr11 | 113614546 | 113614286 | 113614576 | 270 | TGCACCTACTTCCTACACCTTG | ATGGAACTCGCCTATCAGACTT | TRUE | 1139-U15 | 1139-U15 |
| chr11 | 119986114 | 119985862 | 119986146 | 266 | AGCCCTGGGTCTCTACTACC | GACCTGAATCTCTTGTCTTCCC | TRUE | 1139-A16 | 1139-A16 |
| chr12 | 11214250 | 11214222 | 11214336 | 97 | GTGGACCTTCATGCTGGGA | TCCCTTCACTCTGACCCTGAT | TRUE | 1139-A16 | 1139-A16 |
| chr12 | 20903719 | 20903610 | 20904153 | 525 | AGGAATCCCAGCTCCAGTGT | AGCCAAATCCAACTCTTGCC | TRUE | 1139-U17 | 1139-U17 |
| chr12 | 59268262 | 59268238 | 59268690 | 432 | GAGCCATACACATTTCCCTTCA | TGCCAATCCTGGTTCTGGTC | TRUE | 1139-U15 | 1139-U15 |
| chr12 | 62784729 | 62783962 | 62784760 | 780 | AAACCCAGGAAGCACGAAGT | CTTGGTGAGCCTTCTTCATGT | No | 1139-U15 | No Product |
| chr12 | 65268956 | 65268797 | 65269176 | 361 | CAGCATTCTCCCGTCTCAGG | TCCTCCTGGTACTGCCTTCA | TRUE | 1139-U15 | 1139-U15 |
| chr12 | 100042139 | 100041793 | 100042172 | 361 | ACCCTCCCTGTTCTCAGTCA | CGATGCCCATTGTTACCATTC | TRUE | 1139-A9 | 1139-A9 |
| chr12 | 104460023 | 104459944 | 104460329 | 365 | GCTACGAATCAGTGGTTTCTGC | ACTTGCCAGGAAACTTTGGGT | TRUE | 1139-A16 | 1139-A16 |
| chr16 | 88061163 | 88061138 | 88061253 | 96 | CAAGCAGAGCATCGACTCCAA | AAGGGCAGTAGGAGGACGAG | TRUE | 1139-A16,1139-U15 | 1139-A16,1139-U15 |
| chr18 | 58038876 | 58038852 | 58039192 | 321 | GCTCCCTTCATATTGGCACCT | AGCTCCTTGCTTGCATCCAT | TRUE | 1139-A16,1139-U15,1139-U17 | 1139-A16,1139-U15,1139-U17 |
| chr21 | 34399812 | 34399670 | 34400391 | 703 | AGGAGATGAAGCGACTGGTG | ACCAGCTCAGGTTAAAGCCA | No | 1139-A9,1139-U17,UCLA-PCA | No Product |
| chr6 | 117700255 | 117700083 | 117700280 | 179 | TACCCTACAAGCCCAATGCC | TGGTTGGTTCAAGACAGTCAAT | FALSE | UCLA-MET,UCLA-PCA | FALSE |
